# Biopsy‐derived organoids in personalised early breast cancer care: Challenges of tumour purity and normal cell overgrowth cap their practical utility

**DOI:** 10.1002/ijc.35386

**Published:** 2025-02-28

**Authors:** Paul Schwerd‐Kleine, Roberto Würth, Tasneem Cheytan, Laura Michel, Verena Thewes, Ewgenija Gutjahr, Huriye Seker‐Cin, Daniel Kazdal, Sarah‐Jane Neuberth, Vera Thiel, Jonas Schwickert, Tim Vorberg, Jennifer Wischhusen, Albrecht Stenzinger, Marc Zapatka, Peter Lichter, Andreas Schneeweiss, Andreas Trumpp, Martin R. Sprick

**Affiliations:** ^1^ Division of Stem Cells and Cancer German Cancer Research Center (DKFZ) Heidelberg Germany; ^2^ Heidelberg Institute for Stem Cell Technology and Experimental Medicine (HI‐STEM gGmbH) Heidelberg Germany; ^3^ Faculty of Biosciences Heidelberg University Heidelberg Germany; ^4^ Division of Gynecologic Oncology National Center for Tumor Diseases (NCT) Heidelberg Germany; ^5^ Division of Molecular Genetics German Cancer Research Center (DKFZ) Heidelberg Germany; ^6^ Institute of Pathology, Heidelberg University Hospital Heidelberg Germany; ^7^ National Center for Tumor Diseases (NCT) Heidelberg Germany

**Keywords:** breast cancer, genomics, organoids, personalized medicine, quality control

## Abstract

The ability to establish organoids composed exclusively of tumour rather than healthy cells is essential for their implementation into clinical practice. Organoids have recently emerged as a powerful tool to expand patient material in culture and generate modifiable 3D models derived from humans or animal models. For translational research, they enable the creation of model systems for an ever‐increasing number of cell types and diseases. And in personalised medicine, they potentially allow for functional drug testing with high predictive power in certain settings. We found that using biopsy material from untreated, early‐stage primary breast cancer patients poses significant challenges for consistently culturing tumour cells as organoids. Specifically, we observed frequent outgrowth of genetically normal, non‐cancerous epithelial cells. We analysed >100 biopsy samples from early‐stage breast cancer and present our large collection of >70 organoid lines. We also show methods of assessing successful tumour cell culture in a time, and cost‐efficient manner, proving a high rate (>85%) of normal cell overgrowth in early‐stage breast cancer organoids. Finally, we show a number of successful attempts to culture cancer organoids from mastectomy‐derived tissue of advanced, metastatic breast cancer. We conclude that the usefulness of organoids from early breast cancer for translational research and personalised medicine, especially guidance of adjuvant or post‐surgical maintenance therapy, is strongly limited by the low success rate of culturing cancerous cells under organoid conditions.

## Graphical abstract

What's new?

Breast cancer patients often face relapse, sometimes years after initially successful therapy. It would therefore be highly desirable to develop a platform enabling routine assaying of individual efficacy of personalised therapies. Organoids have been proposed as a tool to serve this purpose, but the feasibility of their establishment from biopsy tissue of early breast cancer has not yet been systematically demonstrated. We conclude that the usefulness of organoids for translational research and personalised medicine, especially guidance of adjuvant or post‐surgical maintenance therapy, is strongly limited by the low success rate of culturing cancerous cells under these conditions.

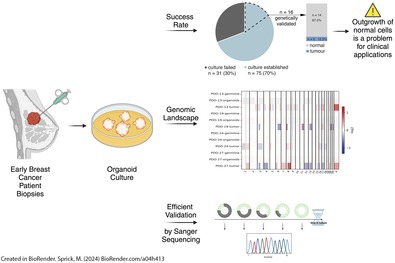

## 1 INTRODUCTION

The advent of personalised treatment strategies has revolutionised patient care in contemporary clinical oncology.^1^ The realisation that patient‐ and cancer‐specific variation in genetic makeup, microenvironment, and other factors profoundly influence disease manifestation and therapy response has caused a paradigm shift towards individually tailoring interventions.^2^, ^3^

In this context, the field was thrilled over the discovery of methods to easily culture organoids—in vitro cellular models that autonomously expand into three‐dimensional structures which more faithfully recapitulate the structural but also functional characteristics and cellular heterogeneity of their organ or tissue of origin.^4^, ^5^ Additionally, organoids present a modifiable system that can be perturbed to turn hypotheses generated from descriptive models into mechanistic understanding.

Because of their aforementioned properties, in some settings organoids were shown to accurately predict the chemosensitivity of patients' tumours. For example, gastrointestinal and pancreatic cancer organoids were used successfully to predict therapeutic response in individual patients.^6^, ^7^

One entity for which the use of organoids holds special promise is early non‐metastatic breast cancer with elevated risk of recurrence. Early non‐metastatic breast cancer patients are often treated with curative intent. Yet, an estimated 20%–30% of patients face metastatic recurrence, where median overall survival is reduced to 2–5 years. As such, assessing therapeutic vulnerabilities of potential drug‐tolerating persister cells after neoadjuvant chemotherapy and surgery could have a crucial impact on the success of post‐surgical maintenance therapy.^8^

Although the possibility of culturing organoids from tissue samples of breast cancer has previously been shown by Sachs et al.,^9^ their study did not involve the clinically relevant case of using tissue from pre‐neoadjuvant needle biopsies. This material would be easily accessible for breast cancer patients, some of whom would, due to their elevated risk of recurrence, frequently benefit from additional therapy personalisation.

Although the idea of utilising organoids in this context holds great promise, there are still largely unaddressed shortcomings to this method.^10^ Most pressing is the need to identify a way to discern organoids derived from neoplastic cells and those originating from “contaminating” healthy epithelial cells. For example, in lung cancer organoids, overgrowth by epithelial airway organoids has been identified as a frequent problem.^11^ Similarly, primary prostate cancer cells were prone to be overgrown by normal prostate epithelial cells,^12^, ^13^ suggesting that cancer cells are often disadvantaged, if co‐cultured with healthy cells. Challenges in efficiently establishing and verifying tumour organoids of high purity are limiting the success of clinical or translational implementation based on results obtained from non‐authenticated tumour organoids.

The question of assessing tumour cell purity in organoids is especially crucial in the context of breast cancer, where no single gene is recurrently mutated to drive oncogenesis and could be used for reliable identification, such as activating KRAS mutations found in 92% of pancreatic cancer patients.^14^ In contrast, breast cancers are driven by an accumulation of multiple, low penetrance mutations. The most frequently mutated genes found in breast cancer are TP53 (41%), PIK3CA (30%), MYC (20%), PTEN (16%), CCND1 (16%), ERBB2 (13%), FGFR1 (11%) and GATA3 (10%).^15^ Of note, some of these driver mutations have been identified in samples of uninvolved adjacent tissue of the mammary gland of breast cancer patients.^16^

Here, we present data on our sizable collective of >75 living organoid lines, established from needle biopsy material of early, non‐metastatic breast cancer. We report success rates, which are severely limited by overgrowth of normal mammary epithelial cells, diminishing tumour purity. Thus, in those cases, organoids do not represent the original tumour faithfully. We detail methods of efficiently identifying pure tumour organoids and report using mastectomy‐derived material from advanced metastatic breast cancer, where success rates were high. These results should be considered when designing future studies on personalised medicine approaches involving organoids from early breast cancer.

## 2 MATERIALS AND METHODS

### 2.1 Patient sample collection

Samples from histologically confirmed early breast cancer were obtained via standard bioptic procedures (14G diameter needle) from consenting patients who were treated with curative intent at the National Centre for Tumour Diseases (NCT), Heidelberg, Germany. Samples were collected as part of the COGNITION trial. Additionally, tissue from palliative mastectomies of metastatic breast cancer patients of all molecular subtypes was collected within the CATCH trial. After collection, samples were immediately transferred into transport medium consisting of CO_2_‐independent medium (Life Technologies), 1% bovine serum albumin (ThermoFisher), Glutamax (ThermoFisher) and Penicillin/Streptomycin (ThermoFisher), and then transported at ambient temperature to our lab at Deutsches Krebsforschungszentrum (DKFZ), also in Heidelberg, Germany.

### 2.2 Organoid culture

Tumour biopsies were dissociated using the Human Tumor Dissociation Kit (Miltenyi). They were transferred to a C Tube (Miltenyi) containing 2.5 mL of a mix of transport medium (for ingredients see above) and a cocktail of digestive enzymes. They were then placed on a gentleMACS Octo (Miltenyi) and dissociated at 37°C with a programme of intermittent mechanical shearing (“37°C h TDK 3”) for 1 h. After digestion, the resulting cell suspension was passed through a strainer with a 100 μm pore size (Greiner Bio‐One), and centrifuged briefly. On ice, the resulting pellet was resuspended in cold BME type II (R&D) at approx. 2 million cells per mL. The resulting suspension was distributed in droplets of 20 μL on a pre‐warmed cell culture dish or multi‐well plate, which is then inverted and left to solidify at 37°C for 5 min. Then the droplets were covered with either the previously published organoid medium^9^ or CSC Medium, which we developed to support the growth of a wide variety of breast cancer cell lines^17^ and incubated (37°C, 5% CO_2_, humidified atmosphere).

The CSC medium recipe was licensed to Miltenyi Biotec, the optimised formulation is available under the name “Breast TumorMACS™ Medium”, only Y27632 has to be added separately according to the manufacturer's instructions, StemMACS™ Y27632, #130‐103‐922.

Passaging was done whenever the organoid spheres started to appear dark in the centre (approximately every 1–2 weeks). Culture medium was aspirated and the organoid droplets were disrupted by repeated pipetting with an appropriate amount of TrypLE Express 1× (ThermoFisher). Dissociation took place at 37°C for 5–10 min. Progress was frequently monitored until digestion was complete. The cell suspension was then taken up in 5–10 mL of transport medium, and centrifuged for 5 min at 300 rpm. Washing was done once with 5 mL of transport medium. Then cells were placed on ice and resuspended in BME, seeded and cultured as described above.

For the three advanced metastatic tumours, tissue was derived from palliative mastectomies. Otherwise, the culturing procedure was the same.

### 2.3 Immunohistochemical analysis, evaluation of tumor cell content

Organoids were lifted out of their cell culture dish, still embedded in their BME matrix and fixed in formalin (4%) overnight. Subsequently, they were dehydrated, embedded in paraffin and sectioned. For immunohistochemistry, the following antibodies were used: anti‐Estrogen Receptor *α* (ThermoFisher RM‐9101‐S1), anti‐progesterone receptor (PR) (ThermoFisher, MA1‐411), anti‐human epidermal growth factor receptor 2 (HER2) (Agilent DAKO, A0485), anti‐human Ki‐67 (Agilent DAKO, GA62661‐2). As a secondary and staining reagent, the Dako Real EnVision Kit (Agilent DAKO, K5007) was used. Tumor cell content in biopsies was evaluated on H&E‐stained sections by a board‐certified pathologist.

### 2.4 Whole genome sequencing

DNA isolation and library preparation were performed by the DKFZ sample processing lab (SPL) for tumour tissue samples, and in our lab for cultured cells. DNA was isolated from the buffy coat of blood sample of patients, and from snap‐frozen material from tumour tissue biopsies. For cultured cells, total genomic DNA was extracted using the Qiaamp micro kit (Qiagen) according to the manufacturer's instructions. Sequencing libraries were prepared using the Illumina TruSeq Nano DNA library preparation kit following standard procedures. Samples were then submitted to the genomics and proteomics core facility of the German Cancer Research Centre for sequencing. Whole genome sequencing (WGS) was performed on an Illumina High Seq X Ten V2.5 (culture samples were pooled and sequenced on 1 lane per sample, while primary samples were sequenced individually on 2 lanes per sample). The sequencing depth ranged from 77× to 84× for tumour and germline samples, and from 39× to 44× for cultured samples. For detailed QC statistics, see Table S1.

### 2.5 Raw data analysis

Raw sequencing reads from WGS were processed by the DKFZ omics and data core facility using their open‐source tool Roddy (https://github.com/TheRoddyWMS/Roddy). To align the sequenced reads, BWA mem (version 0.7.15) was used for DNA and STAR (version 2.5.2b) for RNA against the Genome Reference Consortium human genome (build 37, version hs37d5) concatenated with the bacteriophage Phi X 174 genome, as per the 1000 Genomes Phase 2 assembly. Calling somatic and germline single‐nucleotide variants and small insertions/deletions (indels) on paired tumour‐control samples was based on samtools mpileup and bcftools (version 0.1.19) and Platypus (version 0.8.1). For paired tumour‐control whole‐genome sequencing (WGS) data, ACESeq was used to obtain copy‐number variants (CNVs), estimate tumour ploidy and tumour cell content, and calculate loss of heterozygosity and related homologous recombination deficiency scores. In addition, we used CNVkit to obtain CNVs.^18^

### 2.6 Sanger sequencing

To perform Sanger sequencing, first target mutations were selected from WGS data. Then three sets of primers were designed for each genomic locus using Primer3^19^ and ordered from Merck. Then, total genomic DNA was extracted from pelleted organoids or digested cells from biopsy tissue using the Qiaamp micro kit (Qiagen) according to the manufacturer's instructions. The concentration and purity of the extracted DNA were assessed using a UV–Vis spectrophotometer. Then, the targeted regions of interest were amplified by PCR using the primers previously designed and ordered. PCR reactions were set up in a total volume of 25 μL, containing Q5 High‐Fidelity 2× Master Mix, forward and reverse primers (0.5 μM), template DNA (≥1 ng), and nuclease‐free water. Amplification cycling conditions included an initial denaturation step (98°C, 2 min), followed by 30 cycles of denaturation (98°C, 10 s), annealing (variable temperature, based on primer melting temperature, 20 s), and extension (72°C, 20 s). Lastly, a final extension step was included (72°C, 2 min). Amplified PCR products were then purified using the QiaQuick PCR purification kit (Qiagen) to remove excess primers, nucleotides, and other contaminants. The concentration of the purified PCR products was measured using the Qubit system (ThermoFisher). PCR products were then sent to a provider of overnight Sanger sequencing (TubeSeq Supreme, Eurofins). The resulting ab1 files were then aligned to the genomic regions of interest using Benchling, and the approximate VAF of the mutations of interest was determined from the respective fluorescence intensities.

### 2.7 Publicly available data

To gather datasets focused on breast cancer, we accessed repositories through cBioPortal, leveraging its web interface to execute tailored queries. This platform facilitated the acquisition of comprehensive genomic and clinical data from various breast cancer studies. Subsequently, the acquired datasets were processed and visualised using the statistical software R (v4.3.0). The analysis and presentation of these datasets were conducted using the tidyverse (v2.0.0) and ggplot2 (v3.4.3) packages within R, enabling efficient data manipulation and creation of informative graphical representations. This approach allowed us to explore and communicate key findings derived from publicly accessible breast cancer datasets effectively.

### 2.8 SNP fingerprinting

Genomic data analysis was performed using a custom R script designed for copy‐number alteration detection and sample clustering. Command‐line arguments were utilised to specify input and output file paths, along with optional parameters for data filtering. Sequencing data (fp) and array data (rs) were processed to exclude observations with insufficient coverage and ensure a minimum number of observations per sample. Pairwise correlations between samples were computed based on copy‐number alterations, and samples were clustered using a union‐find algorithm to identify groups exhibiting similar genomic patterns. Results were outputted into text files summarising sample characteristics, correlated samples, and identified clusters. For visualisation purposes, the correlation values between organoid and patient‐derived SNP profiles were plotted as a heatmap using R and the pheatmap package (v1.0.12).

### 2.9 Mouse studies

Animal care and procedures followed the German legal regulations and were previously approved by the governmental review board of the state of Baden‐Württemberg, operated by the local Animal Welfare Office (Regierungspräsidium Karlsruhe) under the license number G‐115/17.

PDOs were injected into the 4th mammary fat pad of 6–8 weeks old female NOD.Cg‐Prkdcscid Il2rgtm1Wjl/SzJ (NSG) mice. Prior to injection, 500,000 cells were resuspended in a 1:1 ratio of sterile PBS and growth factor‐reduced Matrigel (BD).

Tumor growth was monitored by using a digital caliper. Animals were euthanized and tumors were collected when tumors reached a size of ~ 0.5 cm^3^.

### 2.10 Statistical analysis

Statistical analyses were conducted to evaluate factors influencing the success rate of initial organoid cultures derived from needle biopsies of treatment‐naïve, non‐metastatic breast cancer patients. Associations between culturing success and both breast cancer subtype and medium type were tested using chi‐squared tests. The test for breast cancer subtype was performed with three degrees of freedom, while the effect of medium type on success was conducted with one degree of freedom. All statistical analyses were conducted in R (v.4.3.0), with significance set at *p* < .05.

## 3 RESULTS

We established a biobank of 75 living organoid lines, derived using tissue from needle biopsies of treatment‐naïve, non‐metastatic breast cancer patients enrolled in the COGNITION trial run at the National Center for Tumor Diseases (NCT) in Heidelberg, Germany (Table S2). To this end, we took advantage of previously published methods.^9^, ^19^, ^20^ The culture medium used in those publications will be referred to as “organoid medium” in this report. Additionally, we also used our previously reported “CSC medium” which we have demonstrated can sustain growth and cell‐type‐specific properties for a wide variety of breast cancer cell lines.^21^

**FIGURE 1:**
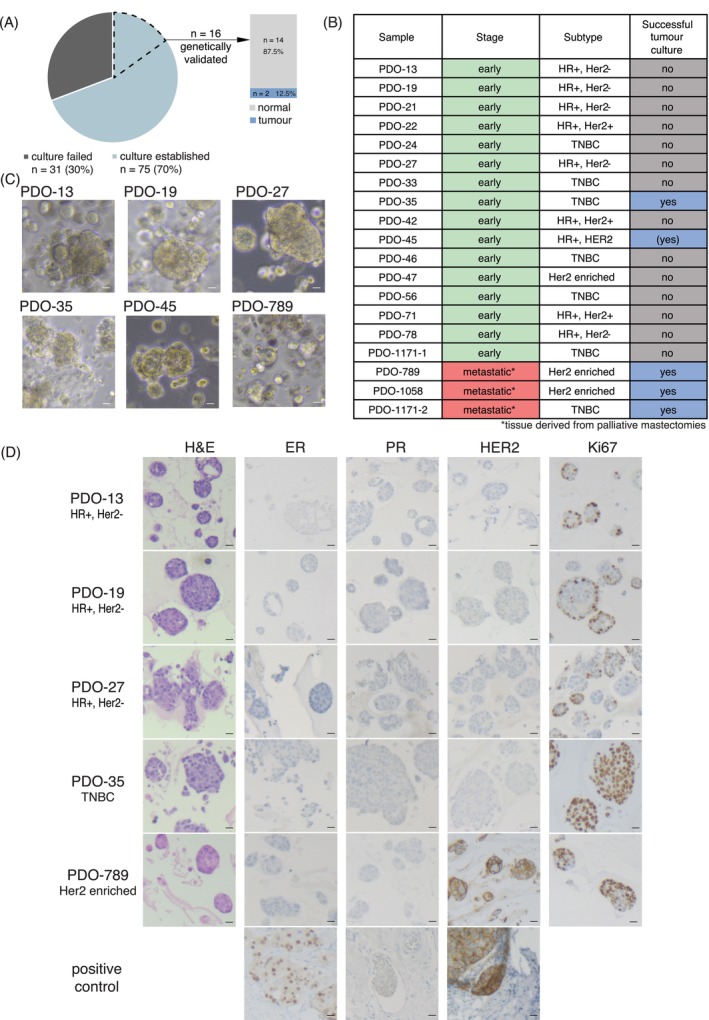
Culturing of organoids from early breast cancer. (A) Success rate of organoid culture establishment. The pie chart shows that 70% (*n* = 75) of biopsy samples successfully established a viable organoid culture, while 30% (*n* = 31) failed. The stacked bar plot indicates the percentage of organoid cultures with a detectable share of cancer cells, with the majority containing a high proportion of tumour cells (87.5%). (B) Overview of samples analysed. This table summarises the breast cancer samples included in the study, detailing the cancer subtype, stage, and whether a successful tumour culture was established. (C) Organoid morphology. Images depict organoids from early breast cancer, typically forming dense, ball‐like structures. Some organoids, like patient‐derived organoid (PDO)‐35, show 2D growth on the dish surface after extended culture. Scale bar = 100 μm. (D) Histology of organoids. Histological images of organoids stained with standard breast cancer markers (H&E, estrogen receptor [ER], progesterone receptor [PR], human epidermal growth factor receptor 2 [HER2], Ki67). The scale bar represents 10 μm.

We achieved a high success rate of 70% for the initial culturing of organoids from needle biopsies of treatment‐naïve, non‐metastatic breast cancer patients (Figure 1A). This was independent of breast cancer subtype (*χ* ^2^ = 2.5025, df = 3, *p*‐value = .4748) and type of medium used (*χ* ^2^ = 0.18091, df = 1, *p*‐value = .6706) (Figure S1 and Table S3). An overview of the subset of samples analysed in this study is presented in Figure 1B. Morphologically, organoids mostly grew in dense, sphere‐like structures (Figure 1C). When left unpassaged for several days (depending on the culture: 5–7 days), organoids started to adhere to the dishes' plastic surface in typical epithelial cobblestone morphology. In 5%–10% of cases with either medium, growth of fibroblasts was observed. However, they either disappeared upon extended passaging or if they remained, the culture was discontinued and discarded. To assess the cellular architecture and expression of typical breast cancer markers (estrogen and progesterone receptor, HER2, and the proliferation marker Ki67), we performed histologic staining on entire organoids still embedded in their growth matrix of BME (Figure 1D). Interestingly, none of the hormone receptor positive samples we analysed showed detectable expression of either estrogen receptor (ER) or PR, which is related to their non‐cancerous nature, as detailed below. In contrast, the HER2‐enriched cancerous PDO‐789 did show strong over‐expression of the HER‐2 receptor in organoids.

**FIGURE 2:**
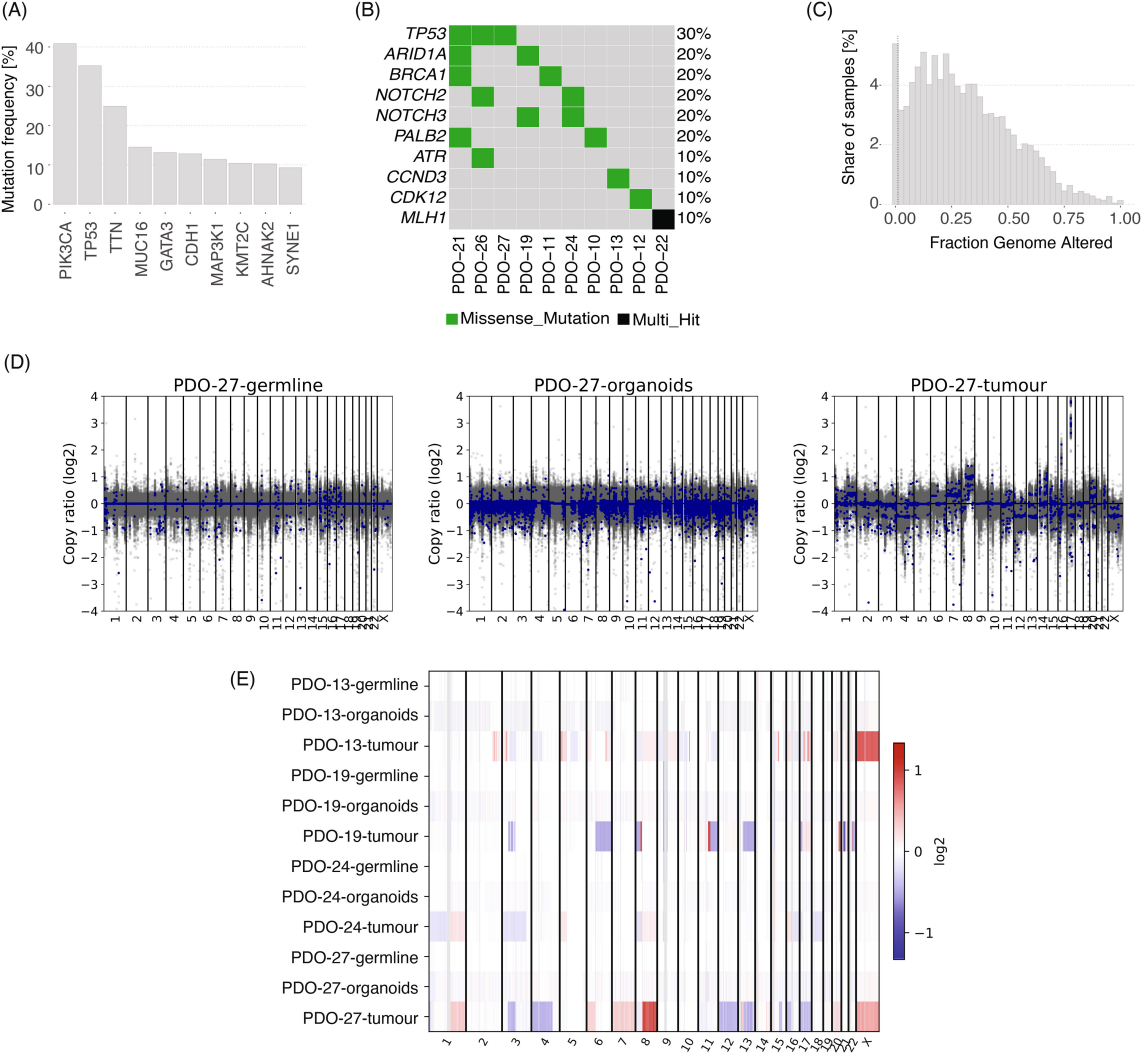
Organoids derived from pre‐treatment biopsies display a normal‐like profile. (A) Mutation frequency of the most frequently altered genes in breast cancer. This bar chart represents the mutation frequency (percentage) of the top 10 most commonly mutated genes in breast cancer. The analysis was conducted using publicly available data from the cBioPortal database, and includes genes such as PIK3CA, TP53, and others that play crucial roles in breast cancer pathogenesis. (B) Most frequently observed gene variants in a selection of organoid lines. The oncoplot displays the most frequently observed mutations in a set of patient‐derived organoid (PDO) lines using the Oncomine Comprehensive Assay v3 gene panel. The graph highlights the prevalence of missense mutations and multi‐hit events across different PDO lines, providing insights into the genetic diversity and common alterations within these organoid models. (C) Histogram of the fraction of the genome affected by structural variants in breast cancer. This histogram illustrates the distribution of the fraction of the genome altered by structural variants across breast cancer samples. The analysis is based on data retrieved from the cBioPortal, showing the extent of genomic instability and the frequency of large‐scale structural alterations for breast cancer. (D) Scatter plot of copy number ratios from CNVkit for PDO‐27. This scatter plot provides a detailed view of the copy number variations (CNVs) observed in the PDO‐27 organoid line, serving as a representative example. The CNVkit analysis reveals significant copy number alterations (CNAs), including marked gains on chromosomes 8, 16, and 17, highlighting regions of genomic amplification that may contribute to tumourigenesis. (E) Heatmap of copy number ratios across multiple samples. The heatmap presents the copy number ratios for different samples, including germline, organoid, and tumour tissues from multiple PDO lines. This visual representation allows for the comparison of CNAs across different conditions, with a notable absence of copy number alterations in the organoid models.

Next, we proceeded with genetic validation of organoids. In breast cancer, the recurrence of characteristic point mutations is limited, as shown by an analysis of publicly available data from cBioportal (Figure 2A). In clinical practice, mutational data are not always available early on, hence in a first attempt to identify tumourous organoids, we ran sequencing based on the Oncomine Comprehensive Assay v3, which is a panel that includes genes frequently mutated in many cancers. However, the results (Figure 2B) did not yield a clear conclusion without prior information on the original tumours' mutation profiles. Caution should be used when interpreting the results of such an approach, due to the testing of multiple variables at the same time, increasing the likelihood of false‐positive discoveries. Contrary to the absence of defining point mutations, almost all breast cancer genomes harbour structural variants, as another visualisation of public genomic data of breast cancer shows (Figure 2C). Thus, the fact that the Oncomine panel did not pick up on any copy number alterations (not shown) hints at samples' genetically normal identity. A clearer picture was obtained by copy number analysis based on WGS data. Each one of the four analysed organoid lines showed a flat copy number profile. All of them were, in contrast to the original tumours, devoid of any high level amplifications or deletions (Figure 2D,E). Additionally, we excluded cross‐contamination and verified culture identity using single‐nucleotide polymorphism (SNP)‐based fingerprinting (Figure S2).

**FIGURE 3:**
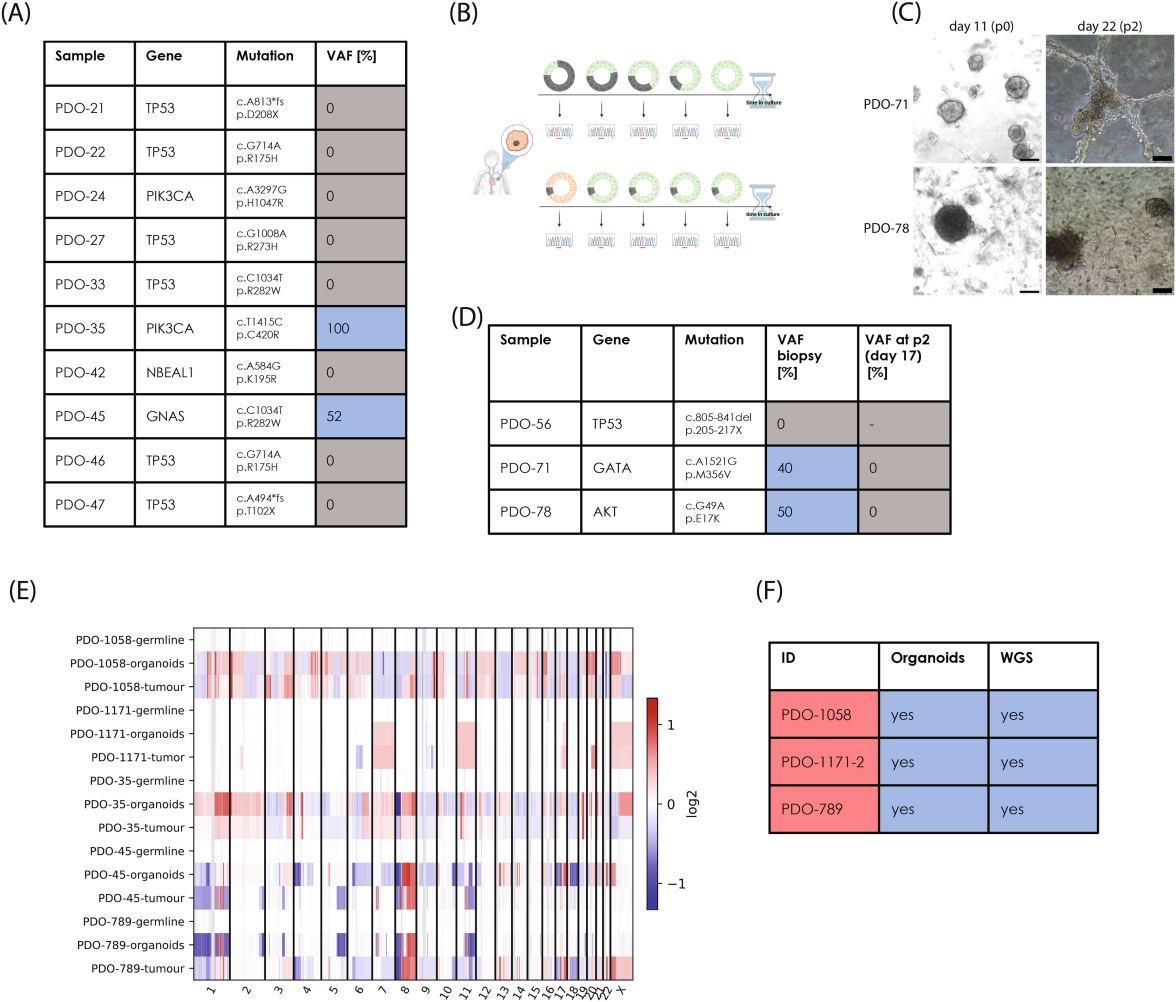
Counter‐selection of cancer cells in organoid cultures derived from early‐stage non‐metastatic breast cancer. (A) Samples used for Sanger sequencing. This panel lists the samples, the specific genes and mutations selected for testing, along with the measured variant allele frequency (VAF) in the biopsy material. (B) Schematic of possible reasons for the rare detection of tumour cells in culture. This diagram outlines potential factors contributing to the rapid counter‐selection of cancer cells in organoid cultures from early‐stage breast cancer. *Created in BioRender. Sprick, M. (2023) BioRender.com/t74w444*. (C) Micrographs of patient‐derived organoid (PDO)‐71 and PDO‐78 organoids. Images show organoids at day 11 (before the first split) and day 22 (5 days after the second split). The scale bar represents 100 μm. (D) Table of samples, genes, and mutations analysed by Sanger sequencing. This table compares VAF measurements at two time points: T0 (biopsy material) and T1 (17 days in culture), illustrating the reduction in detectable tumour mutations during culture. (E) Copy number profiles of organoids. This panel shows the copy number profiles of organoids derived from advanced‐stage (metastatic) tumours and a few early‐stage tumours that still harbour cancer‐associated copy number aberrations, indicating their classification as cancer cells. (F) Validation of tumour identity in advanced‐stage samples. This section confirms that organoids derived from advanced‐stage samples (from palliative mastectomy) were successfully cultured, validated as tumour cells by whole‐genome sequencing (WGS).

To assess whether more than the four tested organoid lines were non‐cancerous, we selected a set of samples for which we had mutation data from WGS of the patients' original tumour and germline DNA. We then proceeded to identify mutations that were present in the original tumour but not in the germline sequence of the respective patient. We only included those that were either previously known oncogenic mutations or were predicted to be highly deleterious. In a second step, we designed primers and ran Sanger sequencing on these loci. Unexpectedly, we found those mutated in only 2 out of 10 tested samples (Figure 3A). Biopsies of the same sampling time point across the entire trial contained tumour cells in >80% of cases (Figure S3), and directly adjacent biopsies of the ones used for organoid cultures had a median tumour cell content of 80% (Table S4). However, given the high rate of normal‐cell outgrowth, we questioned whether the initial biopsy material predominantly consisted of normal cells rather than tumour cells in most samples. This could be attributed to factors like a small tumour volume at the time of diagnosis and biopsy. Alternatively, it is possible that there was an initial detectable fraction of tumour cells that later, in the organoid culture, became diluted due to slow or negligible proliferation, or a combination of both (Figure 3B). To answer this question, it was necessary to benchmark the cultures' tumour cell content against that of the starting biopsy tissue. We thus selected three samples of which we had banked original biopsy material, in addition to the mutational information on these tumours (Figure 3D). We then conducted Sanger sequencing on anticipated mutations directly from a portion of the biopsy material and attempted to cultivate the remainder into organoids. In the case of one sample (PDO‐56), we were unable to identify the expected mutation in the biopsy material, and subsequently, the cells from the other part of the sample we tried to cultivate did not grow. However, for the other two samples, we detected the expected mutations with an estimated variant allele frequency (VAF) of approximately 40% and 50%, respectively. These samples also successfully developed into organoids in culture. By the time of the second passage (17 days in culture), we obtained another DNA sample and performed Sanger sequencing. Notably, even after this brief period in culture, the mutations were undetectable, indicating rapid proliferation of normal cells outcompeting the breast cancer cells.

Finally, we performed WGS on the organoids previously identified as likely tumourous, as well as three additional samples derived from palliative mastectomies of advanced‐staged, metastatic tumours and calculated their copy‐number profiles (Figure 3C). All of them showed CNAs reminiscent of the original tumours, corroborating their cancerous nature. Additionally, tumours arose upon transplantation of organoids into immunodeficient NSG mice (Figure S4), providing functional evidence for their cancerous nature.

## 4 DISCUSSION

Recently, organoids have been promoted as a method to culture and expand material from most solid cancer types, enabling efficient and accurate prediction of an individual tumour's drug sensitivity, and as a tool for preclinical research.^5^ This is applicable in clinical practice for drug‐sensitivity prediction. If introduced successfully, this would be a highly useful tool in the setting of high‐risk early‐stage breast cancer with likely recurrence. Despite the potentially curative setting, some patients do face relapse, and good biomarkers for treatment response are not always readily detected by current means of personalised oncology. Sachs and colleagues previously created a large collection of organoid cultures. However, these were exclusively derived from surgical specimens of lumpectomies, or needle biopsies of metastases. High risk breast cancer patients often undergo neoadjuvant therapy, dictating the need to use pre‐treatment biopsies for any attempts to culture organoids in this setting. Contrastingly, we are unaware of any publication systematically addressing the generation and validation of organoids in this specific setting.

Based on exactly these early‐stage pre‐treatment breast cancer biopsies, we worked towards filling this gap by creating a substantial set of organoid lines and assessing their tumour content by whole‐genome and Sanger sequencing. We found that, despite a very good takerate of the organoid cultures (70%) from this starting material, the percentage of these actually containing breast cancer cells is small (2 out of 16 tested). This was not correlated to the outcome of the patients within the cohort, as the patients from whom the tumourous organoids were derived did not face relapse, while tissue from refractory or relapsing patients did not give rise to tumour organoids (Table S5). This may at first sound surprising, however under optimal growth conditions the faster growth of genetically intact cells has previously been noted for example for airway organoids.^11^ This obviously hinders the use of organoids in personalisation of treatment regimens, because in many cases no useful tumour cultures can be generated. Additionally, each newly established line has to undergo lengthy validation and possibly a subsequent selection and purification process, posing additional practical obstacles. Thus, to make organoids a viable tool to assist clinical decision making, firstly an efficient validation strategy should become standard. This may also be supportive in other tumour entities to ensure high quality of any downstream assay, and should be considered by expert societies when developing guidelines for routine implementation.

In cases where mutation status of the patient is known, we show that targeted Sanger sequencing is a fast and highly cost‐efficient way of assessing tumour content of cultures. In cases where mutation information is unavailable, we suggest utilising a copy‐number alteration detection method, such as low‐coverage whole‐genome sequencing, a copy number chip, or a sequencing panel that is optimised for detection of copy‐number changes. These methods enable precise assessment of tumour content without requiring prior sequencing of both patient germline and tumour.

Beyond that, a more difficult‐to‐tackle challenge will be to increase the applicability of organoids by increasing the efficiency of culturing tumour, but not normal cells. Firstly, there is no widely applicable (surface) tumour marker that could be used for selection, for example by flow sorting. Some surface markers that are more expressed on tumour cells do exist and form the basis of targeted therapies. A well known example, targeting cells expressing Her2 has been standard practice for a long time, and more recently antibody‐drug conjugates targeting Trop2 have entered the arsenal as well.^22^ Another approach that could be considered in addition would be selection by drugs targeting specific mutations. As an example, Nutlin3a has been successfully used to counter‐select p53 wild type cells, while withdrawal of Wnt and R‐spondin selected for APC mutant cells in a model of organoids derived from human intestinal stem cells.^23^ Another interesting approach has been reported by Jacob et al.^24^ In their model of glioblastoma, they used microdissected tissue pieces. This could prove advantageous, since it would avoid disruption of the basement membrane integrity.

A systematic evaluation of these approaches in the setting of breast cancer organoids would be highly desirable in the future.

In summary, our findings show that although organotypic cultures are readily established from biopsy material of pre‐neoadjuvant early breast cancers, the outgrowth of actual tumour cells is highly inefficient. This mandates rigorous quality control and makes their use with current methods essentially impossible for the personalisation of treatment schedules.

## AUTHOR CONTRIBUTIONS

**Paul Schwerd‐Kleine:** Conceptualization; methodology; software; data curation; investigation; validation; formal analysis; visualization; project administration; writing – review and editing; writing – original draft. **Roberto Würth:** Investigation; writing – review and editing. **Tasneem Cheytan:** Investigation. **Laura Michel:** Investigation. **Verena Thewes:** Investigation. **Ewgenija Gutjahr:** Investigation; methodology; writing – review and editing. **Huriye Seker‐Cin:** Writing – review and editing; investigation. **Daniel Kazdal:** Investigation. **Sarah‐Jane Neuberth:** Investigation. **Vera Thiel:** Investigation. **Jonas Schwickert:** Investigation. **Tim Vorberg:** Investigation. **Jennifer Wischhusen:** Investigation; writing – review and editing. **Albrecht Stenzinger:** Writing – review and editing; supervision. **Marc Zapatka:** Supervision. **Peter Lichter:** Supervision; writing – review and editing; funding acquisition. **Andreas Schneeweiss:** Supervision; funding acquisition. **Andreas Trumpp:** Supervision; funding acquisition; writing – review and editing; resources; project administration; conceptualization. **Martin R. Sprick:** Conceptualization; funding acquisition; methodology; project administration; resources; supervision; writing – review and editing.

## FUNDING INFORMATION

We acknowledge support by the BMBF‐funded SATURN^3^ project (01KD2206B; 01KD2206E), the Transluminal‐B and Integrate‐TN (project 70113450) consortia funded by the Deutsche Krebshilfe (both to A.T., P.L. and A.S.), and the Dietmar Hopp Foundation (to A.T.). This project was supported in part by the proof‐of‐concept program of the NCT Heidelberg (to P.L. and A.S.). E.G. was supported by a fellowship of the DKFZ Clinician Scientist Program, supported by the Dieter Morszeck Foundation.

## CONFLICT OF INTEREST STATEMENT

LM has received fees for lectures and travel expenses (congress participation) from: Roche, Eisai, Pfizer, AstraZeneca, Lilly, MSD, Gilead, Daiichi Sankyo. ASt has potential personal conflict of interest as follows: Advisory Board/Speakers Bureau of Agilent, Aignostics, Amgen, Astellas, Astra Zeneca, Bayer, BMS, Eli Lilly, Illumina, Incyte Janssen, MSD, Novartis, Pfizer, Qlucore, QuiP, Roche, Seagen, Servier, Takeda, Thermo Fisher. ASt has received grants from Bayer, BMS, Chugai, Incyte. VThe has reveiced a travel grant from Gilead Sciences. The remaining authors have no conflict of interest.

## ETHICS STATEMENT

Human tissue was obtained based on the study approval protocol by the local institutional review board of Heidelberg University (S‐790/2018). All patients gave written informed consent.

## Supporting information


**Table S1.** Sequencing statistics (WGS).

**Table S2.** Clinical information.

**Table S3.** Take rate of samples cultured in parallel using both media.

**Table S4.** Tumour cell content of adjacent biopsies.

**Table S5.** Follow‐up diagnosis of early stage patients in relation to organoid culture success.

**Data S1.** Supplementary figures.

## Data Availability

The WGS data was uploaded to the EGA under the accession number EGAS50000000605. The code to reproduce the results and figures is publicly available on GitHub (https://github.com/paulemnah/breast_cancer_organoids). Other data that support the findings of this study are available from the corresponding author upon request.

## References

[1] Visvader JE , Rosen JM , Aparicio S . Breast cancer. Cold Spring Harb Perspect Med. 2024;14:a041729. 38692741 10.1101/cshperspect.a041729 PMC11293531

[2] Nolan E , Lindeman GJ , Visvader JE . Deciphering breast cancer: from biology to the clinic. Cell. 2023;186:1708‐1728. 36931265 10.1016/j.cell.2023.01.040

[3] Ye F , Dewanjee S , Li Y , et al. Advancements in clinical aspects of targeted therapy and immunotherapy in breast cancer. Mol Cancer. 2023;22:105. 37415164 10.1186/s12943-023-01805-y PMC10324146

[4] Weeber F , Ooft SN , Dijkstra KK , Voest EE . Tumor organoids as a pre‐clinical cancer model for drug discovery. Cell Chem Biol. 2017;24:1092‐1100. 28757181 10.1016/j.chembiol.2017.06.012

[5] Drost J , Clevers H . Organoids in cancer research. Nat Rev Cancer. 2018;18:407‐418. 29692415 10.1038/s41568-018-0007-6

[6] Vlachogiannis G , Hedayat S , Vatsiou A , et al. Patient‐derived organoids model treatment response of metastatic gastrointestinal cancers. Science. 2018;359:920‐926. 29472484 10.1126/science.aao2774 PMC6112415

[7] Tiriac H , Belleau P , Engle DD , et al. Organoid profiling identifies common responders to chemotherapy in pancreatic cancer. Cancer Discov. 2018;8:1112‐1129. 29853643 10.1158/2159-8290.CD-18-0349 PMC6125219

[8] Pixberg C , Zapatka M , Hlevnjak M , et al. COGNITION: a prospective precision oncology trial for patients with early breast cancer at high risk following neoadjuvant chemotherapy. ESMO Open. 2022;7:100637. 36423362 10.1016/j.esmoop.2022.100637 PMC9808485

[9] Sachs N , de Ligt J , Kopper O , et al. A living biobank of breast cancer organoids captures disease heterogeneity. Cell. 2018;172:373‐386.e10. 29224780 10.1016/j.cell.2017.11.010

[10] Muthuswamy SK , Brugge JS . Organoid cultures for the study of mammary biology and breast cancer: the promise and challenges. Cold Spring Harb Perspect Med. 2024;14:a041661. 38110241 10.1101/cshperspect.a041661 PMC11216180

[11] Dijkstra KK , Monkhorst K , Schipper LJ , et al. Challenges in establishing pure lung cancer organoids limit their utility for personalized medicine. Cell Rep. 2020;31:107588. 32375033 10.1016/j.celrep.2020.107588

[12] Drost J , Karthaus WR , Gao D , et al. Organoid culture systems for prostate epithelial and cancer tissue. Nat Protoc. 2016;11:347‐358. 26797458 10.1038/nprot.2016.006 PMC4793718

[13] Gao D , Vela I , Sboner A , et al. Organoid cultures derived from patients with advanced prostate cancer. Cell. 2014;159:176‐187. 25201530 10.1016/j.cell.2014.08.016 PMC4237931

[14] Bailey P , Chang DK , Nones K , et al. Genomic analyses identify molecular subtypes of pancreatic cancer. Nature. 2016;531(7592):47‐52. 26909576 10.1038/nature16965

[15] Nik‐zainal S , Davies H , Staaf J , et al. Landscape of somatic mutations in 560 breast cancer whole‐genome sequences. Nature. 2016;534:47‐54. 27135926 10.1038/nature17676 PMC4910866

[16] Ronowicz A , Janaszak‐jasiecka A , Skokowski J , et al. Concurrent DNA copy‐number alterations and mutations in genes related to maintenance of genome stability in uninvolved mammary glandular tissue from breast cancer patients. Hum Mutat. 2015;36:1088‐1099. 26219265 10.1002/humu.22845

[17] Würth R , Michel L , Donato E , et al. Circulating tumor cell plasticity determines breast cancer therapy resistance via Neuregulin1/HER3 signaling. Nat Cancer. 2025;6:67‐85. 39753722 10.1038/s43018-024-00882-2 PMC11779641

[18] Talevich E , Shain AH , Botton T , Bastian BC . CNVkit: genome‐wide copy number detection and visualization from targeted DNA sequencing. PLoS Comput Biol. 2016;12:e1004873. 27100738 10.1371/journal.pcbi.1004873 PMC4839673

[19] Untergasser A , Cutcutache I , Koressaar T , et al. Primer3—new capabilities and interfaces. Nucleic Acids Res. 2012;40:e115. 22730293 10.1093/nar/gks596 PMC3424584

[20] Rosenbluth JM , Schackmann RCJ , Gray GK , et al. Organoid cultures from normal and cancer‐prone human breast tissues preserve complex epithelial lineages. Nat Commun. 2020;11:1711. 32249764 10.1038/s41467-020-15548-7 PMC7136203

[21] Dekkers JF , van Vliet EJ , Sachs N , et al. Long‐term culture, genetic manipulation and xenotransplantation of human normal and breast cancer organoids. Nat Protoc. 2021;16:1936‐1965. 33692550 10.1038/s41596-020-00474-1 PMC8221035

[22] Ma Y , Li W , Ma K , Wang T , Feng H . Cell surface markers and their targeted drugs in breast cancer. Curr Protein Pept Sci. 2022;23:335‐346. 35638536 10.2174/1389203723666220530102720

[23] Drost J , van Jaarsveld RH , Ponsioen B , et al. Sequential cancer mutations in cultured human intestinal stem cells. Nature. 2015;521:43‐47. 25924068 10.1038/nature14415

[24] Jacob F , Salinas RD , Zhang DY , et al. A patient‐derived glioblastoma organoid model and biobank recapitulates Interand intra‐tumoral heterogeneity. Cell. 2020;180:188‐204.e22. 31883794 10.1016/j.cell.2019.11.036 PMC7556703

