# Supplementary material for: Biopsy‐derived organoids in personalised early breast cancer care: Challenges of tumour purity and normal cell overgrowth cap their practical utility
Source: Int J Cancer. 2025 Feb 28;156(11):2200–9. doi: 10.1002/ijc.35386 (PMC11970545; doi:10.1002/ijc.35386)
Supplement: Supplementary file 6 — Data S1. Supplementary figures. [file IJC-156-2200-s001.pdf]

Biopsy-Derived Organoids in Personalised Early Breast Cancer Care: Challenges of Tumour Purity and Normal Cell Overgrowth Cap Their Practical Utility

Paul Schwerd-Kleine, Roberto Würth, Tasneem Cheytan, Laura Michel, Verena Thewes, Ewgenija Gutjahr, Huriye Seker-Cin, Daniel Kazdal, Sarah-Jane Neuberth, Vera Thiel, Jonas Schwickert, Tim Vorberg, Jennifer Wischhusen, Albrecht Stenzinger, Marc Zapatka, Peter Lichter, Andreas Schneeweiss, Andreas Trumpp and Martin R. Sprick

Table of Contents

Supplementary Tables (see separate excel files) ***2***

Supplementary Table 1: Sequencing statistics (WGS) **2**

Supplementary Table 2: Clinical information **2**

Supplementary Table 3: Take rate of samples cultured in parallel using both media **2**

Supplementary Table 4: Tumour cell content of adjacent biopsies **2**

Supplementary Table 5: Follow-up diagnosis of early stage patients in relation to organoid culture success **2**

Supplementary Figures ***3***

Supplementary Figure S1: Media conditions, but not subtype, are relevant for organoid culture take rate. **3**

Supplementary Figure S2: Organoid lines are not cross-contaminated **4**

Supplementary Figure S3: Tumour cellularity of adjacent biopsies **5**

Supplementary Figure S4: PDOs of advanced-stage breast cancers are readily tumourigenic *in vivo* **6**

Supplementary Tables (see separate excel files)

Supplementary Table 1**: Sequencing statistics (WGS)**

Supplementary Table 2**: Clinical information**

Supplementary Table 3**: Take rate of samples cultured in parallel using both media**

Supplementary Table 4**: Tumour cell content of adjacent biopsies**

Supplementary Table 5: **Follow-up diagnosis of early stage patients in relation to organoid culture success**

Supplementary Figures

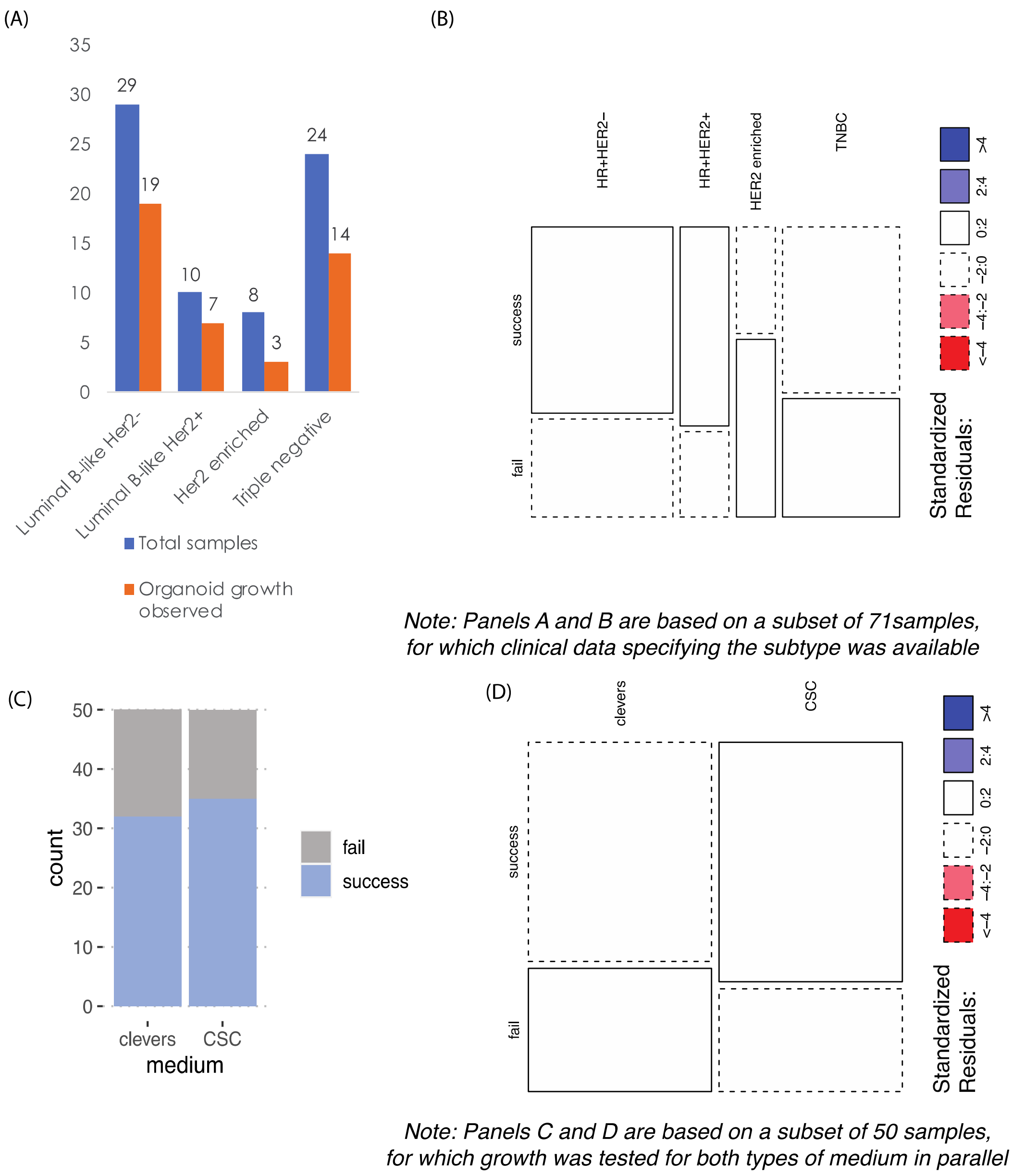

Supplementary Figure S1: Media conditions, but not subtype, are relevant for organoid culture take rate.

A. Bar plot of breast cancer subtypes in our cohort, grouped by total sample number and samples of which a successful organoid culture was derived.

B. Mosaic plot visualising the relationship and standardised residuals of clinical subtype and organoid culture success.

C. Bar plot of culture success rate during our study, divided by growth media. Grouped by total sample number and samples of which a successful organoid culture was derived.

D. Mosaic plot visualising the relationship and standardised residuals of clinical subtype and organoid culture success.

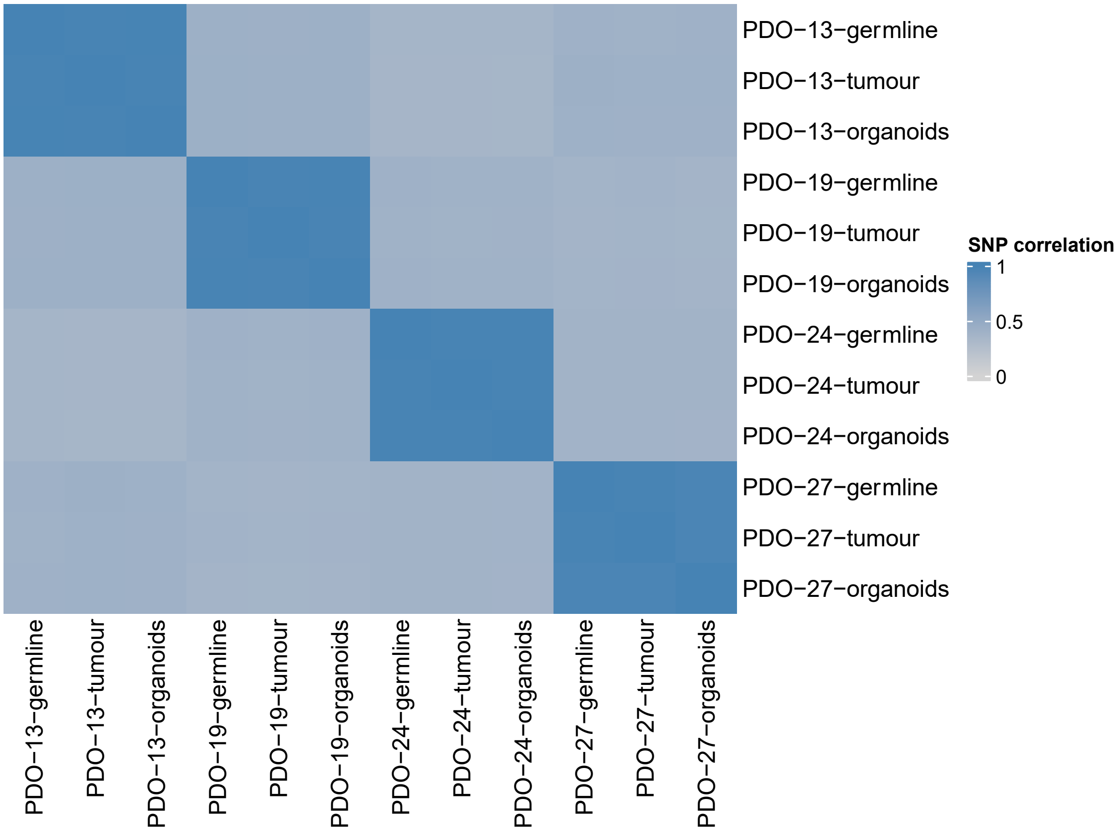

Supplementary Figure S2: Organoid lines are not cross-contaminated

Heatmap of Pearson correlation of single-nucleotide variants in germline, tumour and organoid derived whole-genome sequences of four patients.

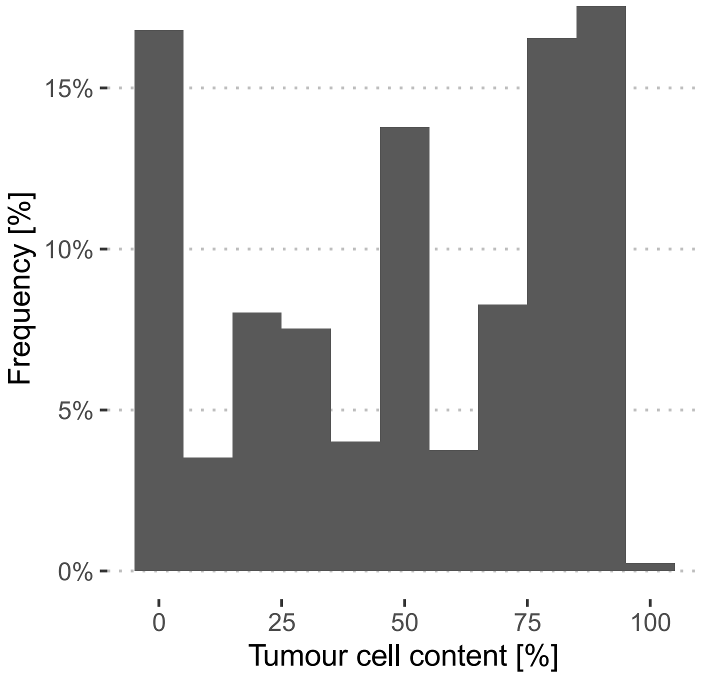

Supplementary Figure S3: Tumour cellularity of adjacent biopsies

Distribution of tumor cell content (%) in biopsy samples, showing the frequency of samples with different levels of tumor cell content. The tumour cell content was annotated by the examining pathologist. The assessed biopsies were taken from adjacent tissue of those used for culturing organoids.

(A)

Sample	Subtype	Stage	Number of tumours/ total mice	Median time to endpoint
PDO-13	HR+, Her2-	early	0/3	--
PDO-19	HR+, Her2-	early	0/6	--
PDO-24	TNBC	early	0/6	--
PDO-27	HR+, Her2-	early	0/6	--
PDO-789	Her2 enriched	advanced/ metastatic	6/6	5.5 weeks
PDO1058	Her2 enriched	advanced/ metastatic	6/6	4 weeks
PDO-1171-1	TNBC	early	0/3	--
PDO-1171-2	TNBC	advanced/ metastatic	3/3	3 weeks

(B)

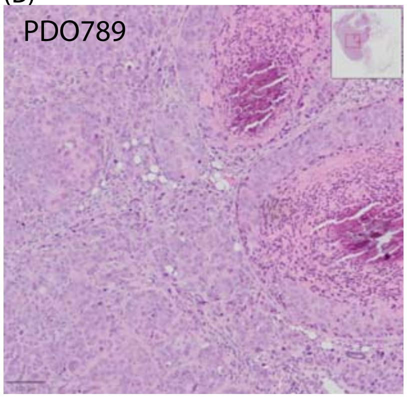

(C)

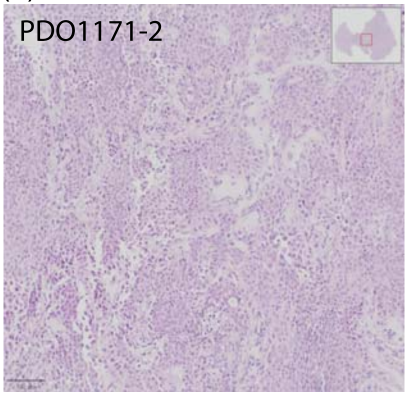

Supplementary Figure S4: PDOs of advanced-stage breast cancers are readily tumourigenic *in vivo*

A. Table detailing samples used for *in vivo* tumourigencity test via orthotopic transplantation. All mice injected with PDOs from advanced-stage cancers developped tumours, while non of the early stage PDOs did. The early stage PDOs all displayed a normal-like genotype as well (Fig. 2).
B. H&E staining of a representative region of a PDX from PDO789.
C. H&E staining of a representative region of a PDX from PDO1171-2.
note: we did not reserve tissue of PDX 1058 for H&E staining.
